# Prediction of risk factors and outcomes of neonatal acute kidney injury

**DOI:** 10.1007/s40620-021-01130-x

**Published:** 2021-09-01

**Authors:** Kumail AlGadeeb, Mostafa Qaraqei, Rahma Algadeeb, Hassan Faqeehi, Abdulrahman Al-Matary

**Affiliations:** 1 Neonatology Department, Maternity and children hospital, Alhasa, Saudi Arabia; 2 Neonatology Department, King Fahad Medical City, Riyadh, Saudi Arabia; 3 Postgraduate Center for Preventive Medicine, Alhasa, Saudi Arabia; 4 Department of Nephrology, Children’s Hospital, King Fahad Medical City, Riyadh, Saudi Arabia

**Keywords:** AKI, Prevalence, Risk factors, Infants, Neonatal

## Abstract

**Introduction:**

Neonatal Acute kidney injury (AKI) is an underestimated morbidity in the neonatal intensive care unit (ICU). However, there is a paucity of information about risk factors, outcomes, and possible preventive measures to limit its occurrence.

**Aim:**

This study aimed to determine the prevalence of neonatal AKI in a neonatal ICU. Data obtained from this study will help to better understand current local practices and investigate possible preventive strategies.

**Materials and methods:**

Charts from January 2011 to December 2018 were reviewed. Neonates less than 2 weeks old who depended on intravenous fluid as a nutrition source for at least two days were included.

**Results:**

Overall, the eight-year prevalence of neonatal AKI in the neonatal ICU was 19.6%, and severity was distributed as follows: stage 1 (46.2%), stage 2 (26.5%), and stage 3 (27.3%). Caffeine administration before 29 weeks’ gestational age significantly decreased the incidence of neonatal AKI. The incidence of neonatal AKI was independently associated with death (odds ratios (OR) = 7.11, *P* < 0.001) and extended length of hospital stay (OR = 2.47, *P* < 0.001). In the multivariate regression model, vancomycin (AOR = 1.637, *P* < 0.004), loop diuretics (AOR = 2.203, *P* < 0.001), intraventricular hemorrhage (AOR = 2.605, *P* < 0.001), surgical intervention (AOR = 1.566, *P* < 0.008), mechanical ventilation (AOR = 1.463, *P* < 0.015), and dopamine administration (AOR = 2.399, *P* < 0.001) were independently associated with neonatal AKI.

**Conclusion:**

Neonatal AKI occurred in one-fifth of the study population in a neonatal ICU. Outcomes can be improved by identifying high-risk infants and cautiously monitoring kidney function.

## Introduction

Neonatal Acute kidney injury (AKI) is a commonly underestimated morbidity in the neonatal intensive care unit (ICU) [1], 2. Individuals with neonatal AKI are at risk of death and chronic kidney disease [3]. There is no standard definition of neonatal AKI and thus no precise data regarding the actual incidence of neonatal AKI in the neonatal ICU (NICU) [4]. The most commonly used definition in studies researching neonatal AKI is the one recommended by the Kidney Disease Improving Global Outcomes (KDIGO), which uses serum creatinine and urine output to define and stage severity [5]. The KDIGO definition is the preferred definition because it is directly related to mortality and length of hospital stay [6]. Sepsis is the most common causative factor of neonatal AKI, accounting for more than three-quarters of all neonatal AKI cases in the NICU [7]. In Thailand, published epidemiological data showed a neonatal AKI prevalence of 6.3% in admitted babies; sepsis was the most common risk factor in these infants [8]. A recent Egyptian study published in the Saudi Journal of Kidney Disease and Transplantation stated that more than half of all neonatal AKI cases in their units were sepsis-induced [9]. Exposure to inotropes, non-steroidal anti-inflammatory drugs (NSAIDs), and other nephrotoxic medications may contribute to AKI in neonates [10]. KDIGO recommends using theophylline to prevent neonatal AKI in patients suffering from birth asphyxia [11]. Additionally, drugs that increase renal blood flow, such as dopamine, may protect the kidneys [12].

The AWAKEN study is a large multicenter, multinational observational cohort study that enrolled more than 2000 infants from 24 neonatal units. This study describes neonatal AKI incidence by gestational age as a U-shaped distribution that peaks from 22 to 29 weeks of gestation and peaks again in fetuses after 36 weeks of gestation, which is likely related to the critical condition of those fetuses. According to the AWAKEN study, nearly one-third of critically ill neonates have AKI, and those who do are four times more likely to die compared with normal controls [13]. Very interestingly, a local single-center study performed in western Saudi Arabia showed that one out of two babies admitted to the ICU has neonatal AKI, and the mortality rate among infants is 28% [14].

We conducted a case–control study evaluating neonatal AKI in NICUs to estimate the prevalence in a tertiary center in Riyadh, Saudi Arabia and to identify the common risk factors leading to this morbidity.

## Methods

We reviewed 5406 neonatal database charts, including all babies admitted to the NICU from January 2011 until December 2018. We included all admitted neonates with at least two recorded creatinine readings beyond the third day of life and only those who required intravenous fluid as their primary source of nutrition because this indicated the severity of their condition. Only neonates admitted to our NICU before 14 days of life were included in this study. We excluded all babies with bilateral kidney abnormalities, those with lethal genetic syndromes, and those who died in the first two days of life. The modified neonatal KDIGO definition was used to categorize neonates according to neonatal AKI severity. We depended chiefly on creatinine level increases because our records were lacking or it was difficult to track urine output for most of the neonates.

### Statistical analysis method

The data analyses were performed using the Statistical Packages for Social Sciences (SPSS) version 20, Armonk, NY: IBM Corp. All categorical variables had been presented as numbers and percentages. The associations between independent factors and the dependent variable were calculated using the Chi square test. Logistic regression was used to calculate crude odds ratios (ORs) and associated 95% confidence intervals (CIs) for the association between neonatal AKI and the likelihood of death. A multivariate logistic model was run to account for potential confounding variables, and the findings are reported as the adjusted OR and adjusted parameter estimate. A *P* value of less than 0.05 was considered statistically significant.

### Data collection

All data were collected using the Ian Neonatal Database with the aid of the CORTEX hospital information system. Data were extracted to an Excel sheet, and all creatinine readings were reviewed. The included data assessed demographic information including gestational age, birth weight, and gender; maternal risk factors including pregnancy-induced hypertension, membrane rupture more than 18 h before delivery, use of antenatal dexamethasone, maternal diabetes, and multiple gestations; perinatal risk factors including mode of delivery, Apgar score at 5 min, asphyxia, and hypothermia with a temperature less than 36 °C; postnatal risk factors including respiratory distress syndrome (RDS), culture-proven sepsis, intraventricular hemorrhage (IVH), hemodynamically significant patent ductus arteriosus (PDA), and necrotizing enterocolitis (NEC); NICU risk factors such as mechanical ventilation; iatrogenic interventions such as central venous line and use of umbilical arterial catheter; surgical interventions; and use of nephrotoxic medications including aminoglycosides, vancomycin, ceftazidime, amphotericin B, loop diuretics, NSAIDs, and inotropes. We wanted to assess a possible protective effect of caffeine and dopamine, as well as being an infant of a mother with pre-eclampsia, so we included these values in the collected data and analysis. Outcomes were reported as mortality, stage, and onset of neonatal AKI, and length of hospital stay.

The modified KDIGO definition for AKI in newborns classifies the severity of neonatal AKI into stages based on changes in serum creatinine and reductions in urine output. We used this definition because it is independently associated with mortality and length of stay (LOS). We largely depended on creatinine levels to define neonatal AKI.

• Stage 1–Increase in serum creatinine by ≥0.3 mg/dL (≥26.5 µmol/L) within 48 hours
• Stage 2–Increase in serum creatinine by 150% to <200% from previous trough levels
• Stage 3–Increase in serum creatinine by 200% to <300% from previous trough levels or serum creatinine ≥2.5 mg/dL (221 µmol/L)

In patients who had two creatinine level peaks, which indicates multiple kidney injury events, the neonatal AKI stage was classified according to the highest level recorded, and the timing of the initial peak was considered the onset. Any neonate with fewer than two creatinine level measurements beyond the third day of life was excluded due to insufficient data. We subdivided our included population into three groups according to gestational age: less than 29 weeks, 29–37 weeks, and more than 37 weeks’ gestational age.

## Results

**Fig. 1:**
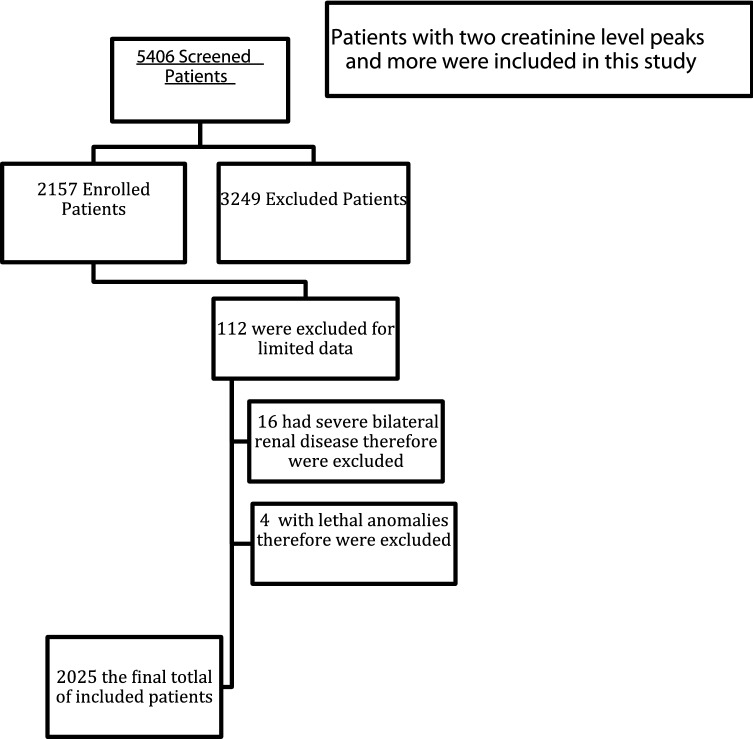
Patient selection flow chart

A total of 2,157 neonates were enrolled in this study, and 2,025 were included in the final sample [1116 boys (59.1%) and 909 girls (40.9%)] (Fig. 1). Of these neonates, 396 were diagnosed with neonatal AKI, yielding an overall prevalence of 19.6%. The onset of neonatal AKI was most likely to occur during the first week of life (39%). Nearly half of neonatal AKI cases were classified as stage 1 (mild) (46%), followed by stage 3 (27.3%) and stage 2 (26.5%). Table 1 presents the infants’ characteristics in accordance with neonatal AKI. Based on the results, the incidence was almost equally distributed among the three gestational age groups with a slightly higher incidence in those between 29 and 37 gestational weeks (34.6%), followed by less than 29 gestational weeks (34.1%) and term pregnancies (31.3%), but U-shaped peaks in regard to birth weight were evident. Neonatal AKI was reported in nearly half of neonates (46%) with a birth weight of less than 1.5 kg but in less than one-third of neonates (32.3%) with a birth weight of more than 2.5 kg.

**Table 1:** Characteristics of infants according to AKI^(*n*=2025)^

Infant characteristics	AKI *N* (%)(*n*=396)	No AKI *N* (%)(*n*=1629)
Gender		
Male	234 (59.1)	882 (54.1)
Female	162 (40.9)	747 (45.9)
Birth weight		
< 1 kg	103 (26.0)	230 (14.1)
1–1.5 kg	81 (20.5)	400 (24.6)
> 1.5–2 kg	84 (21.2)	615 (37.8)
> 2.5 kg	128 (32.3)	384 (23.6)
Gestational age		
Term	124 (31.3)	448 (27.5)
29–37 weeks	137 (34.6)	957 (58.7)
< 29 weeks	135 (34.1)	224 (13.8)
Mode of delivery		
Vaginal	238 (60.3)	1139 (69.9)
Cesarean	157 (39.7)	490 (30.1)
Apgar score at 5 min		
< 5	13 (03.3)	33 (02.0)
≥ 5	383 (96.7)	1596 (98.0)

Maternal characteristics	AKI *N* (%)(*n*=396)	No AKI *N* (%)(*n*=1629)	*P* value^§^
Multiple gestation	107 (27.0)	430 (26.4)	0.801
Diabetes	09 (02.3)	51 (03.1)	0.370
Hypertension	38 (9.6)	214 (14.8)	0.024**
Steroids	136 (18.6)	595 (81.4)	< 0.001**

*AKI* Acute Kidney Injury

^§^*P* value has been calculated using the Chi square test

**Significant at *P* < 0.05 level

When comparing maternal characteristics in accordance with the presence of neonatal AKI, mothers with hypertension (10.3% vs. 14.8%) and those receiving antenatal steroids (18.6% vs 81.4%) were less likely to have neonates with AKI. Conversely, the distributions of mothers with multiple gestations and diabetes were similar in both groups.

Neonates with AKI were more likely to use nephrotoxic medications including vancomycin, ceftazidime, amphotericin B, loop diuretics, and NSAIDs (*P* < 0.001). Neonatal AKI occurred more frequently in very sick neonates who required mechanical ventilation or were on epinephrine and dopamine infusions (Table 3), those who had respiratory distress syndrome, necrotizing enterocolitis, intraventricular hemorrhage, culture-proven sepsis, or in those who underwent surgical intervention. Surprisingly, there was no significant association with hypoxic-ischemic encephalopathy or low Apgar score, admission with hypothermia, or the use of aminoglycosides**.** The presence of an umbilical arterial line or central venous catheter was observed more often in the neonatal AKI group (Table 2).

**Table 2:** Risk factors according to AKI (*n*=2025)

Factor	AKI *N* (%) (*n*=396)	No AKI *N* (%) (*n*=1629)	*P* value^§^
Gender			
Male	234 (59.1)	882 (54.1)	0.076
Female	162 (40.9)	747 (45.9)	
Birth weight			
≤ 2.5 kg	268 (67.7)	1245 (76.4)	< 0.001**
> 2.5 kg	128 (32.3)	384 (23.6)	
Gestational age			
Term	124 (31.3)	448 (27.5)	< 0.001**
29–37 weeks	137 (34.6)	957 (58.7)	
< 29 weeks	135 (34.1)	224 (13.8)	
Apgar score at 5 min			
< 5	13 (03.3)	33 (02.0)	0.132
≥ 5	383 (96.7)	1596 (98.0)	
Nephrotoxic medications			
Aminoglycoside	316 (79.8)	1349 (82.8)	0.159
Vancomycin	242 (61.1)	455 (27.9)	< 0.001**
Ceftazidime	54 (13.6)	83 (05.1)	< 0.001**
Amphotericin B	39 (09.8)	33 (02.0)	< 0.001**
Loop diuretics	124 (31.3)	145 (08.9)	< 0.001**
NSAIDs	82 (20.7)	108 (06.6)	< 0.001**
Central line	215 (54.3)	450 (27.6)	< 0.001**
Umbilical atrial catheter	118 (29.8)	176 (10.8)	< 0.001**
Admission temperature < 36	192 (48.5)	841 (51.6)	0.262
Hypoxic ischemic encephalopathy	05 (01.3)	19 (01.2)	0.874
Necrotizing enterocolitis	51 (12.9)	85 (05.2)	< 0.001**
Patent Ductus Arteriosus	88 (22.2)	203 (12.5)	< 0.001**
Respiratory distress syndrome	201 (54.3)	687 (47.6)	0.021**
Premature Rupture of Membrane > 18 h	42 (10.6)	154 (09.5)	0.487
Intraventricular hemorrhage	47 (11.9)	37 (02.3)	< 0.001**
Surgical intervention	149 (37.6)	234 (14.4)	< 0.001**
Mechanical ventilation	252 (63.6)	523 (32.1)	< 0.001**
Positive sepsis	113 (28.5)	212 (13.0)	< 0.001**
Epinephrine infusion	85 (21.5)	60 (03.7)	< 0.001**

*AKI* Acute Kidney Injury; *NSAIDs* Non-steroidal anti-inflammatory drugs; *GA* Gestational age

^§^*P* value has been calculated using the Chi square test

**Significant at *P* < 0.05 level

When comparing the clinical outcomes by AKI status (Table 4), we found that the mortality rate (*P* < 0.001) and extended LOS (*P* < 0.001) were significantly associated with neonatal AKI. Moreover, the non-survival rate was significantly higher in stage 3 neonatal AKI (*P* < 0.001) whereas an extended LOS (*P* < 0.001) and dopamine use (20.8%) were inversely associated with neonatal AKI.

The incidence of neonatal AKI was significantly lower in preterm (less than 29 weeks of gestation) neonates who were administered caffeine (*P* < 0.043) but not in neonates who received caffeine but were born after more than 29 weeks gestation (*P* < 0.335).

In the regression analysis assessing clinical outcomes, the crude odds ratio indicated that the odds of having neonatal AKI were seven times higher in patients who did not survive compared to those who did survive (AOR = 7.11, *P* < 0.001); after adjustments, the odds were two times higher (AOR = 2.12, *P* < 0.001). The odds of having neonatal AKI in patients who had an extended hospital stay were two times higher than in those without an extended stay (AOR = 2.47, *P* < 0.001); however, after adjustment, this difference was not statistically significant (AOR = 0.84, *P* < 0.359) (Table 3).

**Table 3:** Clinical outcomes by AKI status ^(*n*=2025)^

Factor	AKI		*P* value^§^	AKI Stage				*P* value^§^
	AKI *N* (%) (*n*=396)	No AKI N (%) (*n*=1629)		0 N (%) (*n*=1629)	1 N (%) (*n*=183)	2 N (%) (*n*=105)	3 N (%) (*n*=108)	
Mortality								
Non-survival	122 (30.8)	97 (06.0)	< 0.001**	97 (06.0%)	42 (23.0)	27 (25.7)	53 (49.1)	< 0.001**
Survival	274 (69.2)	1532 (94.0)		1532 (94.0)	141 (77.0)	78 (74.3)	55 (50.9)	
Extended LOS	156 (39.4)	339 (20.8)	< 0.001**	339 (20.8)	72 (39.3)	41 (39.0)	43 (39.8)	< 0.001**
Dopamine	182 (46.0)	209 (12.8)	< 0.001**	209 (12.8)	65 (35.5)	56 (53.3)	61 (56.5)	< 0.001**
Caffeine								
GA ≥ 29 weeks	45 (32.8)	276 (28.8)	0.335	276 (28.8)	27 (38.0)	09 (31.0)	09 (24.3)	0.363
GA < 29 weeks	115 (85.2)	206 (92.0)	0.043**	206 (92.0)	56 (84.8)	37 (86.0)	22 (84.6)	0.247

*AKI* Acute Kidney Injury; *LOS* Length of Hospital Stay

^§^*P* value has been calculated using the Chi square test

**Significant at *P* < 0.05 level

The evaluation of risk factors associated with neonatal AKI determined that taking vancomycin during pregnancy was associated with significantly higher odds of the newborn developing neonatal AKI (AOR = 1.637, *P* < 0.004); a similar relationship was observed between loop diuretics and neonatal AKI (AOR = 2.203, *P* < 0.001). We also found that infants who had intraventricular hemorrhage (AOR = 2.605, *P* < 0.001), surgical intervention (AOR = 1.566, *P* < 0.008), mechanical ventilation (AOR = 1.463, *P* < 0.015), and who were on dopamine infusions (AOR = 2.399, *P* < 0.001) all had a significantly increased risk of neonatal AKI. Other variables included in the model, such as low birth weight (LBW), ceftazidime, amphotericin B, NSAIDs, central line, umbilical atrial catheter, necrotizing enterocolitis, patent ductus arteriosus, respiratory distress syndrome, positive sepsis, epinephrine, caffeine at GA > 29 weeks, hypertension, steroids, and gestational age were not significantly associated with neonatal AKI (Tables 4, 5).

**Table 4:** Prediction model for the clinical outcome ^(*n*=2025)^

Factor	CRUDE OR (95% CI)	*P* value	AOR (95% CI)	*P* value
Mortality				
Non-survival	7.11 (5.29–9.56)	< 0.001**	2.12 (1.38–3.26)	0.001**
Survival	Ref		Ref	
Extended LOS	2.47 (1.96–3.13)	< 0.001**	0.84 (0.59–1.21)	0.359

*AKI* Acute Kidney Injury; *NSAIDs* Non-steroidal anti-inflammatory drugs; *AOR* Adjusted Odds Ratio

Crude Odds Ratio is presented for Mortality rates and extended length of Hospital Stay. Odds ratios were adjusted in the model such as; mortality, Extended LOS, birth weight, vancomycin, ceftazidime, amphotericin B, loop diuretics, NSAIDs, central line, umbilical atrial catheter, hypertension, Necrotizing Enterocolitis, Patent Ductus Arteriosus, Respiratory Distress Syndrome, Intraventricular Hemorrhage, Surgical Intervention, Mechanical Ventilation,Positive Sepsis, Epinephrine, Dopamine, Caffeine, hypertension, steroids, and gestational age

**Significant at *P* < 0.05 level

**Table 5:** Multivariate regression analysis predicting the effect of a risk factor to AKI ^(*n*=2025)^

Factor	AOR	95% CI	*P* value^§^
Low Birth weight	0.858	0.607–1.214	0.387
Maternal medication			
Vancomycin	1.637	1.175–2.280	0.004**
Ceftazidime	0.866	0.549–1.366	0.522237
Amphotericin B	1.302	0.702–2.416	0.402
Loop diuretics	2.203	1.565–3.101	< 0.001**
NSAIDs	1.403	0.882–2.231	0.153
Central Line	1.116	0.783–1.589	0.544
Umbilical atrial catheter	1.062	0.700–1.612	0.777
Necrotizing enterocolitis	1.039	0.633–1.708	0.879
Patent ductus arteriosus	1.009	0.708–1.437	0.962
Respiratory distress syndrome	1.067	0.729–1.561	0.739
Intraventricular hemorrhage	2.605	1.465–4.631	0.001**
Surgical intervention	1.566	1.124–2.183	0.008**
Mechanical ventilation	1.463	1.076–1.987	0.015**
Positive sepsis	1.041	0.730–1.484	0.825
Epinephrine	1.416	0.866–2.314	0.165
Dopamine	2.399	1.676–3.435	< 0.001**
Caffeine at GA < 29 weeks	0.909	0.611–1.353	0.639
Hypertension	0.776	0.508–1.186	0.241
Steroids	0.780	0.558–1.090	0.145
gestational age	1.112	0.747–1.656	0.600

*AKI* Acute Kidney Injury; *NSAIDs* Non-steroidal anti-inflammatory drugs; GA-Gestational age; *AOR* Adjusted Odds Ratio

Crude Odds Ratio is presented for Mortality rates and extended length of Hospital Stay. Odds ratios were adjusted in the model such as; mortality, Extended LOS, birth weight, vancomycin, ceftazidime, amphotericin B, loop diuretics, NSAIDs, central line, umbilical atrial catheter, hypertension, Necrotizing Enterocolitis, Patent Ductus Arteriosus, Respiratory Distress Syndrome, Intraventricular Hemorrhage, Surgical Intervention, Mechanical Ventilation, Positive Sepsis, Epinephrine, Dopamine, Caffeine, hypertension, steroids, and gestational age

**Significant at *P* < 0.05 level

**Fig. 2:**
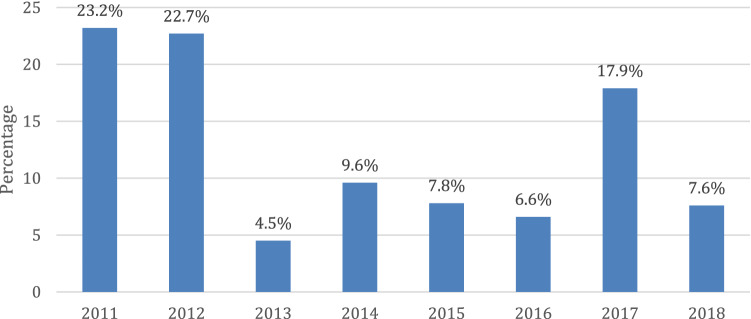
Incidence of AKI per year

**Fig. 3:**
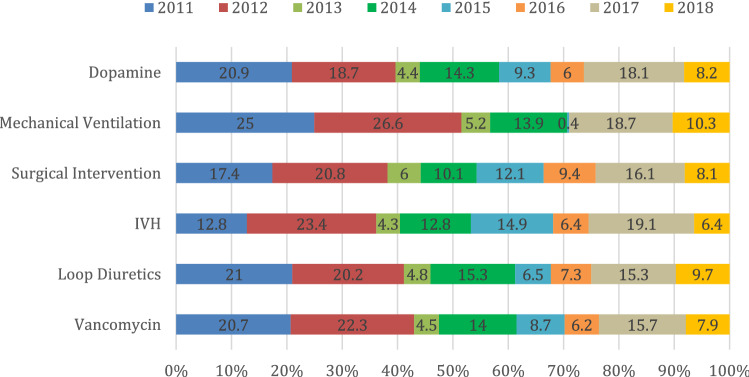
Prevalence of AKI in regards to Independent Significant

The incidence of neonatal AKI was substantially higher during 2011 (23.2%) and 2012 (22.7%), followed by 2017 (17.9%); conversely, it was relatively lower during 2013 (4.5%). To investigate this finding, we plotted the prevalence of neonatal AKI in regards to independent significant factors per year (Fig. 2). Based on the results, dopamine, mechanical ventilation, surgical intervention, intraventricular hemorrhage, loop diuretics, and vancomycin were shown to be independent significant factors. The incidence of neonatal AKI among all these factors was less notable during the period from 2013 to 2016 compared with 2011 and 2012 (Fig. 3).

## Discussion

Neonatal AKI is an underestimated event that has not been sufficiently investigated in infants. This might be related in part to the lack of a standard definition of neonatal AKI. Moreover, there is a lack of diagnosing stage 1 neonatal AKI among neonatologists [1], 2. One in five neonates admitted to the NICU and enrolled in this study had neonatal AKI, corresponding to an overall eight-year prevalence of 19.6%. Curiously, more than two-thirds of cases occurred in preterm babies (68.7%). This incidence is consistent with values reported in studies by Elmas et al. [15] and Koralkar et al. [21]. These papers reported neonatal AKI prevalence rates of 20% and 18% among neonates, respectively. Nearly half (46.2%) had stage 1 mild neonatal AKI, whereas 26.5% had stage 2 neonatal AKI and 27.3% had stage 3 neonatal AKI. Other studies have reported higher incidences. For example, Shalaby et al. [14] conducted a one-year prospective cohort study that reported a neonatal AKI incidence of 56%. Similarly, Lee et al. [17] reported a neonatal AKI incidence of 56%, among whom 30%, 17%, and 9% of the total patients were classified as stage 1, stage 2, and stage 3 neonatal AKI, respectively. The differences compared with our incidence can be explained by the larger sample size and number of years included in our study (eight years vs one year). In the United States, (13, 20–21) the prevalence of neonatal AKI ranged from 30 to 54%, which was higher than our study findings. By contrast, our result was higher than the study of Malla et al. [21], which reported a prevalence of 11.6%.

The eight-year study period, larger sample size, and a wide variety of investigated risk factors allowed us to evaluate differences in local practices and subsequent outcomes. We were able to observe the effect of different birth weights on the occurrence of neonatal AKI. Two incidence peaks were observed: one in small neonates less than 1.5 kg and another peak in those more than 2.5 kg. The AWAKEN study noted a U-shaped occurrence regarding gestational age (less than 29 weeks and above 36 weeks), which was not identified in our research. These results were likely obtained because lower weight is also associated with more risk factors of prematurity such as RDS, NEC, and IVH.

Although LBW might be detrimental to neonatal AKI, the prevalence and its effect on neonatal AKI in such neonates remain uncertain. In this study, the prevalence of LBW among neonatal AKI cases was high (67.7%). In the univariate analysis, we found that LBW was significantly associated with neonatal AKI; however, this relationship was no longer statistically significant in the multivariable analysis. This result is consistent with the study by Shalaby et al. [14] who reported that compared to infants without neonatal AKI, infants with neonatal AKI had a lower birth weight, which increased the chance of mortality. Similarly, this effect was noted in extremely low birth weight infants in whom neonatal AKI was shown to reduce survival before the post menstrual age (PMA) of 36 weeks [15].

In general, the causality of any risk factor should be closely examined because the kidney injury might be a cause or effect of, or association with, these risk factors. There have been conflicting reports regarding the risk factors concomitant to neonatal AKI. For example, Shalaby et al. [14] reported that gestational age, perinatal depression, and the Clinical Risk Index for Babies (CRIB II) score were associated with an increased risk of neonatal AKI. However, Lee et al. [17] indicated that high-frequency ventilation support, the presence of patent ductus arteriosus, lower gestational age, and inotropic agent use were independently associated with neonatal AKI. Ghobrial et al. [9] noted that a history of maternal illness, low body temperature, sepsis, prematurity, and respiratory distress can contribute to the development of neonatal AKI (in neonates). Criss et al. [19] reported that there were no significant risk factors related to neonatal AKI, including gender, age, birth weight, cardiac comorbidities, PDA, IVH, diuretic use, birth to diagnosis time, vasopressors, bacteremia, Bell’s criteria, and antibiotics. In this study, we report that the use of vancomycin and loop diuretics, intraventricular hemorrhage, surgical intervention, mechanical ventilation, and dopamine administration were independently associated with neonatal AKI. There tended to be a lower incidence of neonatal AKI in infants born to mothers with pre-eclampsia, although this relationship was not statistically significant. Some of the associated risk factors may be related to the poor perfusion of the kidneys, such as being critically sick, or the use of epinephrine, which might also affect decreasing fractional renal blood flow. We anticipated that dopamine may have affected reducing the severity of neonatal AKI in poorly perfused neonates; however, it showed a paradoxical effect, which can be explained by the association between poorly perfused kidneys and systemic hypotension. Although the use of mechanical ventilation is significantly associated with the general sickness of neonates, high mean airway pressure is known to decrease preload and thus kidney perfusion. Substantial morbidities related to any surgical intervention place neonates at risk of kidney insult, including sepsis, hypotension, stress, and the underlying pathology. Vancomycin and loop diuretics are known as nephrotoxic medications, and the significant relationship between loop diuretic use and neonatal AKI found in our study may be linked to the practice of administering furosemide with low urine output.

We used the modified KDIGO classification to investigate neonatal AKI, as have most recently published studies. This classification is independently linked to mortality and extended LOS; moreover, it was a feasible tool to use in our unit.

Despite differences compared with other reports, we reported mortality rates of 30.8% among infants with neonatal AKI. We further asserted that the incidence of neonatal AKI was independently associated with death and extended hospital stay. The non-survival rate was significantly higher in infants with higher neonatal AKI stages. However, after adjusting for confounders, only mortality rates remained independently associated with neonatal AKI. These results are consistent with a study published by Shalaby et al. [13], which concurred that the overall mortality rate was 28.3% and that neonatal AKI was significantly associated with mortality but not with the length of hospital stay. A similar finding has been noted by Criss et al. [18], who reported that mortality rates among neonates with AKI were 35.9%. They further surmised that neonates with AKI had higher mortality and a greater chance of death, but the effect of LOS on survivors did not reach statistical significance, which agreed with our results. In the UAE, [20] the prevalence of mortality rates was moderately high (58%), and mortality rates were the only independent significant factor associated with the presence of neonatal AKI. A high incidence of neonatal AKI was shown by Elmas et al. [14], who revealed that 61.9% of neonates died prematurely due to the presence of neonatal AKI. By contrast, a paper by Carmody et al. [19] reported the lowest incidence rate of death among babies with neonatal AKI (8%); they further extrapolated that neonatal AKI was associated with increased mortality and LOS.

Our study suggests that administering caffeine to preterm infants can be beneficial and can prevent potential neonatal AKI. Apparently, our results were in line with the potential benefits of administering caffeine to preterm infants; we showed that caffeine administration in infants born preterm at less than 29 weeks of gestation was significantly more likely to decrease the chances of having neonatal AKI. This result was in agreement with the study of Harer et al. [15], who observed that neonatal AKI occurred less frequently among neonates who received caffeine than among those who did not. In their multivariable model, the administration of caffeine remained associated with reduced odds of developing neonatal AKI; specifically, for every five neonates exposed to caffeine, one case of neonatal AKI was prevented. Lee et al. [16] reported that maternal pre-eclampsia was a protective factor before the post-menstrual age of 36 weeks whereas El-Badawy et al. [9] indicated that continuous positive airway pressure ventilation may have a protective effect against neonatal AKI. These reports were not congruent with our outcome.

We noticed that our cases of neonatal AKI peaked in 2011 and 2012 and then showed a dramatic decrease in incidence, as did the independent risk factors, which might reflect the effect of the implementation of a sepsis quality control project at that time. Although sepsis was not an independent risk factor in our study, it was directly linked to most of our independent risk factors. Moreover, sepsis was a common risk factor in other studies in the literature.

A limitation of this study is its retrospective design; thus, insufficient data may have resulted in an underestimation of the prevalence of risk factors. Moreover, we faced difficulties in classifying some of the neonates with high creatinine results, who lacked applicable urine output volumes or in those with low creatinine values but with daily urine outputs fitting the KDIGO definition of neonatal AKI. We noticed that frequent creatinine readings were taken if the neonates had neonatal AKI whereas such readings were less frequent if the results were normal, which might not accurately reflect the actual incidence in the unit. We depended solely on serum creatinine measurements due to the lack of urine output data, which also supports the definition of neonatal AKI. The AWAKEN study reported that one-third of their neonatal AKI cases would have been missed if they had not considered urine output measurements. Moreover, using serum creatinine might not be the optimal marker to reflect kidney function as creatinine level takes a long time to rise after injury, especially with the paucity of muscle mass in small infants.

Conversely, the strengths of our study are the inclusion of a large number of neonates over an eight-year study period and the investigation of variable risk factors, outcomes, and possible preventive measures. We also emphasized how changes in practices may affect different risk factors and outcomes. Having a single-center study highlights the effect of local practices, but a prospective multicenter study would provide a more generalized idea and better scope on risk factors and outcomes.

## Conclusion

During the eight-year study period, neonatal AKI was reported in one-fifth of the evaluated neonates in a neonatal ICU. Vancomycin, loop diuretics, intraventricular hemorrhage, surgical intervention, mechanical ventilation, and dopamine administration are independent factors associated with neonatal AKI. Moreover, neonatal AKI was significantly associated with death and extended hospital stay. Conversely, administering caffeine at GA < 29 weeks was a protective factor against neonatal AKI. Neonatal AKI outcomes can be improved by identifying high-risk infants and cautiously monitoring kidney function. These recommendations are useful for pediatric physicians specializing in neonatal AKI care.

## Abbreviations

AKI: Acute kidney injury
ICU: Intensive care unit
KDIGO: Kidney Disease Improving Global Outcomes
NSAIDs: Non-steroidal anti-inflammatory drugs
LOS: Length of stay
IVH: Intraventricular hemorrhage
NEC: Necrotizing enterocolitis
RDS: Respiratory distress syndrome
