# Correction to: Prediction of risk factors and outcomes of neonatal acute kidney injury

**DOI:** 10.1007/s40620-021-01155-2

**Published:** 2021-09-15

**Authors:** Kumail AlGadeeb, Mostafa Qaraqei, Rahma Algadeeb, Hassan Faqeehi, Abdulrahman Al-Matary

**Affiliations:** 1 Neonatology Department, Maternity and Children Hospital, Alhasa, Saudi Arabia; 2 Neonatology Department, King Fahad Medical City, Riyadh, Saudi Arabia; 3 Postgraduate Center for Preventive Medicine, Alhasa, Saudi Arabia; 4 Department of Nephrology, Children’s hospital, King Fahad Medical City, Riyadh, Saudi Arabia

## Correction to: Journal of Nephrology 10.1007/s40620-021-01130-x

The co-authors and their affiliations are omitted in the published article.

The correct list of author names and their affiliations are copied below:

Kumail AlGadeeb^**1**^, Mostafa Qaraqei^**2**^, Rahma Algadeeb^**3**^, Hassan Faqeehi^**4**^, Abdulrahman Al-Matary^1,*^

**1.** Neonatology department, Maternity and children hospital, Alhasa, Saudi Arabia

**2.** Neonatology department, King Fahad Medical city, Riyadh, Saudi Arabia

**3.** Postgraduate center for preventive medicine, Alhasa, Saudi Arabia

**4.** Department of Nephrology, Children’s hospital, King Fahad Medical City, Riyadh, Saudi Arabia

*Corresponding author, amatary@yahoo.com

The original article has been corrected.

